# A cholinergic eligibility trace facilitates amygdala plasticity in flavour avoidance learning

**DOI:** 10.64898/2026.07.02.734913

**Published:** 2026-10-16

**Authors:** Weston Fleming, Jordan L. Pauli, Kentaro K. Ishii, Marta Trzeciak, Cassidy T. Burke, Sekun Park, Ruohe Liu, Adam G. Gordon, Larry S. Zweifel, Richard D. Palmiter, Garret D. Stuber

## Abstract

When an animal feels ill after consumption of a novel food, it rapidly learns to avoid this food in the future, a form of learning termed conditioned flavour avoidance (CFA). Postingestive malaise often occurs long after consumption, necessitating a neural mechanism that can facilitate plasticity between temporally distant events. Neuromodulators, acting through G-protein-coupled receptors (GPCRs) that can influence neuronal excitability on extended timescales, may facilitate this process. The projection of parabrachial (PB) *Calca* neurons to the central amygdala (CeA) promotes formation of CFA. Here, we demonstrate that these neurons overlap with a PB population that releases the neuromodulator acetylcholine (ACh) in the CeA during both consumption of a novel solution and subsequent visceral malaise, consistent with a role in CFA acquisition. Calcium imaging in brain slices reveals that ACh widely activates CeA neurons and enhances glutamatergic responsivity on a timescale consistent with CFA learning. CRISPR-Cas9-mediated genetic knockdown and optogenetics demonstrate that ACh from PB facilitates CFA acquisition. Loss of ACh signalling to CeA blocks key signatures of CFA-associated plasticity. Together, these data reveal that cholinergic input from PB to central amygdala is a critical neuromodulatory signal that links activity over long timespans to facilitate associative learning.

